# Gambling symptoms, behaviors, and cognitive distortions in Japanese university students

**DOI:** 10.1186/s13011-019-0230-5

**Published:** 2019-11-13

**Authors:** Kengo Yokomitsu, Takanobu Sakai, Tomonari Irie, Jun Tayama, Hirokazu Furukawa, Mika Himachi, Junichiro Kanazawa, Munenaga Koda, Yoshihiko Kunisato, Hirofumi Matsuoka, Takuhiro Takada, Fumito Takahashi, Takahito Takahashi, Kaori Osawa

**Affiliations:** 1 Ritsumeikan University, 2-150 Iwakura-cho, Ibaraki, Osaka, 567-8570 Japan; 2 Konan Women’s University, 6-2-23 Morikita-machi, Higashinada-ku, Kobe, Hyogo 658-0001 Japan; 3 Hokusho University, 23 Bunkyodai, Ebetsu, Hokkaido 069-8511 Japan; 4 Waseda University, 2-579-15 Mikajima, Tokorozawa, Saitama, 359-1164 Japan; 5 Naruto University of Education, 748 Nakashima, Takashima, Naruto-cho, Naruto, Tokushima 772-8502 Japan; 6 Tokai Gakuen University, 2-901 Nakahira, Tenpaku-ku, Nagoya, Aichi 468-8514 Japan; 7 Health Sciences University of Hokkaido, 1757 Kanazawa, Tobetsu-cho, Ishikari-gun, Hokkaido 061-0293 Japan; 8 Tokushima University, 1-1 Minamijosanjimacho, Tokushima, 770-8502 Japan; 9 Senshu Univiersity, 2-1-1 Higashimita, Tama-ku, Kawasaki, Kanagawa 214-8580 Japan; 10 Shinshu University, Nishi-Nagano 6-ro, Nagano, 380-8544 Japan; 11 University of Miyazaki, 1-1 Gakuenkibanadai-nishi, Miyazaki, 889-2192 Japan; 12 Konan University, 8-9-1 Okamoto, Higashinada-ku, Kobe, Hyogo 658-8501 Japan

**Keywords:** Gambling, University students, Japan, Cognitive distortion

## Abstract

**Background:**

This study was conducted to investigate the relationship between symptoms of gambling problems, gambling behaviours, and cognitive distortions among a university student population in Japan ages 20 to 29 years. We aimed to address the gap in knowledge of gambling disorders and treatment for this population.

**Methods:**

Data were obtained from 1471 Japanese undergraduate students from 19 universities in Japan. Descriptive statistics and hierarchical multivariate regression analysis were used to investigate whether the factors of gambling cognitive distortions would have predictive effects on gambling disorder symptoms.

**Results:**

Results indicated that 5.1% of the participants are classifiable as probable disordered gamblers. The bias of the gambling type to pachinko and pachislot was unique to gamblers in Japan. Of the students sampled, 342 self-reported gambling symptoms via the South Oaks Gambling Screen. Hierarchical multivariate regression analysis indicated that one domain of gambling cognitive distortions was associated significantly with gambling symptoms among the 342 symptomatic participants: gambling expectancy (*β* = 0.19, *p* < .05). The multivariate model explained 47% of the variance in the gambling symptoms.

**Conclusion:**

This study successfully contributed to the sparse research on university student gambling in Japan. Specifically, our results indicated a statistically significant relationship between gambling cognitive distortions and gambling disorder symptoms. These results can inform the development of preventive education and treatment for university students with gambling disorder in Japan. The report also describes needs for future research of university students with gambling disorder.

## Background

The life-time prevalence of gambling disorder (previously designated as pathological gambling) in people who speak English and other European languages has been reported as 0.8–1.2% [24]. By contrast, the prevalence rate (6.13%) of gambling disorder among college and university students, according to meta-analysis of previous research, has been reported as higher than that of adults [20]. Earlier studies established age as a risk factor of gambling disorder [11]. Actually, Johansson et al. [11] suggested that younger than 29 years old appeared to be a significant risk factor for gambling disorders. Although college and university students might find some benefits to gambling, such as financial gain and emotional excitement [3], most of them face various adverse results from gambling behaviour such as financial harm, relationship disruption, emotional or psychological distress, and reduced performance at part-time work or studies [16]. Moreover, among university students, gamblers have more academic problems than non-gamblers, such as decreased worse GPA, spending 3 hours or more using the computer for non-academic purposes, and spending 3 hours or fewer studying [14]. In Japan, one reason students withdraw from college is poor academic performance (14.5% of all reasons) [19]. These academic difficulties might increase the drop-out risk or leave of absence from university classes, and affect student’s lives outside of school. Notably, of Japanese young people who dropped out of university, the rate of people who had been working as full-time permanent employees for 3 years or above was 1.7%, compared with 60% of university and graduate school students who had been working as full-time permanent employment [27]. Based on the description above, the severe impact of gambling disorder on university students’ health and lives evinces the need for effective, timely treatments to reduce gambling problems and behaviours among university students. Additionally, preventive education and information should be offered to university students at risk of developing a gambling disorder, such as social gamblers and non-disordered gamblers [2, 10].

The improvement of empirical knowledge on gambling disorder among university students is extremely important for providing such evidence-based treatment and preventive education [1]. In Japan, few reports examine disordered gamblers in university student populations. In fact, information related to gambling disorder, gambling behaviours and cognitive distortions in this population is unavailable. For instance, the prevalence ratio of gambling disorder for Japanese university students has never been reported. Without this information, it is difficult to understand the magnitude of this issue within Japan, nor in comparison globally or with other countries. Accordingly, it is necessary to evaluate the conditions of gambling disorder and cognitive distortions in the Japanese university student populations. Therefore, this study sought to ascertain the gambling symptoms, behaviours, and cognitive distortions in a university student sample in Japan, ages 20 to 29 years. We also aimed to determine if, in this novel population, we could accept previous findings that cognitive distortions would predict gambling disorder symptoms.

Cognitive distortions among gamblers show a significant predictive effect on gambling disorder in high school students and first-year college students in the United Kingdom [4]. Cognitive behavioural therapy, the most effective psychological intervention for gambling disorder, has helped gamblers to improve their associated behaviour and cognitive distortions [9]. Gamblers’ cognitive distortions are various, typically including illusions of control and gambling expectancy. For example, the illusion of control describes a gambler’s belief in his or her probability of personal success that is unjustifiably high [15]. The correction of these cognitive distortions uses a strategy called ‘cognitive restricting’. Disordered gamblers who acquired skills to identify and correct their gambling cognitive distortions would be able to reduce their gambling behaviours effectively. Earlier studies have shown gambling cognitive distortions are a predictive factor of gambling disorder among college and university students [8]. Since there is insufficient evidence of gambling among Japanese student, it remains unclear whether gambling cognitive distortions can similarly predict gambling disorder in the target population.

Therefore, we sought first to ascertain the gambling symptoms, types, and cognitive distortions in a university student sample in Japan, aged up to 29 years. Moreover, we sought secondly to ascertain whether the factors of cognitive distortions would have predictive effects on gambling disorder symptoms.

## Method

### Ethics

The study protocol was approved by the Ethics Committee at the Graduate School of Education and Human Development, Nagoya University (ID: 14–568).

### Participants

A paper questionnaire was delivered to 2286 Japanese university students ages 20 and older from April 1, 2015 through March 30, 2016. In Japan, people aged under 20 years old in horse racing and under 18 years old in pachinko and pachislot are prohibited by law. Participants were asked to participate in the study irrespective of their gambling frequency. Questionnaires were distributed to students in nine national and public universities (Aichi University of Education, Nagasaki University, Nagoya University, Naruto University of Education, Shinshu University, University of Miyazaki, University of Tsukuba, University of the Ryukyus, and Utsunomiya University) and nine private universities (Aoyama Gakuin University, Health Sciences University of Hokkaido, Hokusho University, Kanto Gakuen University, Konan University, Senshu University, Tokai Gakuen University, Toyo Eiwa University, and University of Human Environments). Of the 1539 (67.3%) responses received, 68 responses were deemed invalid and removed: 50 responses had missing data; 18 responses were from subjects 30 years or older. In Japan, because students who enrolled at university in late 20 or 30 years old were very few in number, we excluded these students in this study [21]. Participant characteristics are presented in Table 1

**Table 1:** Descriptive statistics for the sample

	Total	Gambler
	*N*=1,471	*N*=342
	%	%
Age (mean (SD))	20.91 (1.27)	21.15 (1.56)
Sex		
Male	45.4	69.0
Female	54.6	31.0
Monthly income		
< 10,000 JPY	4.4	2.3
10,000–19,999	3.4	2.6
20,000–29,999	5.2	2.9
30,000–39,999	7.8	7.6
40,000–49,999	5.2	3.5
50,000–59,999	14.3	16.7
60,000–69,999	7.6	6.4
70,000–79,999	8.9	9.1
80,000–89,999	10.3	10.8
90,000–99,999	3.2	3.5
100,000–199,999	27.0	30.1
≧ 200,000	3.1	4.4

*SD* standard deviation, *JPY* Japanese yen

### Measures

#### Demographics

Participants were asked questions about gender, age, and monthly income, including scholarship and/or financial assistance from parents.

#### Gambling types and behaviours

Participants were asked to indicate the types of gambling they participated in during the prior year. Participants were asked to indicate the number of days they had gambled during the prior month (“How many days did you gamble in the last month?”), the amount of money they had spent on gambling (“How much did you spend on gambling in the last month? You need not count the income and expenditures on gambling but the money invested.”), and the duration of gambling play (“How much time have you spent gambling?”).

#### Gambling symptoms

The South Oaks Gambling Screen (SOGS [17]) is a 20-item self-report measure that assesses gambling symptoms. It produces a score ranging from 0 to 20 (the authors of the scales do not score Item 9 [17]). A total score of 1–2 indicates non-problem gambling (“non-problem gamblers”) and 3–4 indicates at-risk gambling (“at-risk gamblers”). A score of 5 or more indicates probable gambling disorder (“disordered gamblers”). The South Oaks Gambling Screen–Modified Japanese version (SOGS-J) [23], which has been shown to have high sensitivity (100.0%) and specificity (94.5%); thus it is appropriate for screening Japanese individuals for gambling disorder [25]. The Cronbach’s alpha for this study was acceptable (*α* = .73).

#### Cognitive distortions

The Gambling Related Cognitions Scale (GRCS [22]) assesses five gambling-related cognitions: illusion of control, predictive control, interpretative bias, gambling expectancy, and perceived inability to stop gambling. The Japanese version of the GRCS (GRCS-J) [29] is a 23-item questionnaire designed to measure gambling-related cognitions. As with the GRCS, participants responded using a seven-point Likert scale to indicate the extent to which they agreed with the values expressed in each item. Higher scores indicated a higher number of cognitive distortions. The GRCS-J has excellent internal consistency (*α* = .94) and good convergent validity with the SOGS-J (*r* = .61) [29]. In this study, the total scale demonstrated high internal consistency (*α* = .97).

### Procedure

#### Questionnaire distribution

At all universities, the questionnaires were distributed to the university students in the classroom. The completed questionnaires were put in collection boxes at each university. A questionnaire package with a consent form and an information sheet was distributed to the participants to complete on their own time. We explained that those who did not consent to participation in this study would not be placed at any disadvantage.

### Statistical analysis

Analyses were conducted using SPSS software (IBM SPSS Statistics package 22.0; SPSS Inc., Chicago, IL., USA; R 3.3.2 R Core Team, 2016, Vienna, Austria). First, descriptive statistics for demographic data, gambling types, symptoms, and cognitive distortions were presented as means and standard deviations (SD). Secondly, a hierarchical multivariate regression analysis was conducted to examine the effects of gambling cognitive distortions on gambling disorder symptoms, adjusting student’s sex, age, income, and gambling behaviours (the number of days they had gambled during the prior month, the amount of money spent on gambling during the prior month, and duration of gambling play). Responses from 1471 complete surveys were analysed with all incomplete responses removed. No missing values were included in the analyses as only the completed responses were considered. For all tests in this study, significance (two-tailed) was inferred for *p* < .05.

## Results

### Demographic characteristics, gambling types, symptoms, and cognitive distortions

Tables 2 and 3 show participants’ gambling symptoms, types of gambling, and cognitive distortions. According to the SOGS-J scores, of the 1471 participants, 14.8% (*n* = 218) were identified as “non-problem gamblers”, 3.3% (*n* = 49) were identified as “at-risk gamblers”, and 5.1% (*n* = 75) were identified as “disordered gamblers”. Nearly a quarter (*n* = 342, 23.2%) of the participants self-identified as gamblers, with 63.7% scoring as non-problem gamblers, 14.3% scoring as at-risk gamblers and 21.9% scoring as disordered gamblers. The percentages for male non-problem gamblers, at-risk gamblers, and disordered gamblers were 61.9% (*n* = 135), 67.3% (*n* = 33), and 90.7% (*n* = 68), respectively, among the 342 gamblers. In addition, the total and subscale GRCS scores were, 54.64 and 8.23–15.08, respectively.

**Table 2:** Participants’ gambling types and frequencies

	Gambler	Once a year	Once a month	Once a week
	N = 342			
	% (n)	% (n)	% (n)	% (n)
Slot machines (not online)	32.7 (112)	10.8 (37)	11.4 (39)	10.5 (36)
Pachinko	29.2 (100)	10.8 (37)	8.8 (30)	9.6 (33)
Lottery (loto, numbers, etc.)	19.3 (166)	16.7 (57)	1.8 (26)	0.9 (13)
Mahjong	13.2 (145)	7.3 (25)	4.1 (14)	1.8 (16)
Horse races	12.0 (141)	7.6 (26)	1.5 (25)	2.9 (10)
Motorboat races	14.7 (116)	2.6 (29)	1.5 (25)	0.6 (12)
Toto (sport betting)	13.2 (111)	3.2 (11)	0	0
Casino (not online)	12.9 (110)	2.9 (10)	0	0
Keirin (bicycle races)	11.5 (115)	1.2 (24)	0.3 (21)	0
Others	16.2 (121)	4.1 (14)	1.5 (25)	0.6 (12)

**Table 3:** Participants’ gambling behaviors, symptoms, and cognitive distortions

	Gamblers	Disordered gamblers	At-risk gamblers	Non-problem gamblers
	*n* = 342	*n* = 75	*n* = 49	*n* = 218
	Mean (*SD*, skewness)	Mean (*SD*)	Mean (*SD*)	Mean (*SD*)
Gambling behaviors during past 1 month				
Number of days	2.85 (5.98, 2.68)	7.95 (8.43)	4.04 (6.14)	0.83 (3.20)
Amount money (JPY)	16293.86 (65504.46, 11.41)	43704.00 (117016.48)	27853.06 (75329.63)	4265.60 (19567.78)
Gambling history (month)	15.73 (25.55, 2.13)	29.48 (28.87)	25.90 (28.58)	8.71 (20.57)
Gambling symptoms				
South Oaks Gambling Screen	2.77 (2.43, 1.45)	6.77 (1.74)	3.45 (0.50)	1.24 (0.43)
Gambling cognitive distortions				
Gambling Related Cognitions Scale				
Total	54.64 (27.74, 0.67)	79.67 (25.94)	64.71 (23.00)	43.77 (22.48)
Illusion of control	8.23 (4.96, 1.06)	11.47 (5.38)	10.00 (4.86)	6.72 (4.12)
Predictive control	15.08 (8.01, 0.50)	20.72 (7.52)	18.86 (7.40)	12.29 (6.89)
Interpretative bias	10.41 (6.36, 0.60)	15.25 (5.62)	12.76 (5.49)	8.22 (5.66)
Gambling expectancies	9.58 (5.55, 0.71)	14.64 (5.12)	11.10 (5.02)	7.49 (4.48)
Inability to stop gambling	11.34 (7.06, 1.05)	17.59 (7.25)	12.00 (5.89)	9.04 (5.82)

*SD* standard deviation, *JPY* Japanese yen

### Preliminary and correlational analyses

For all variables, we calculated means, standard deviation, and skewness (Table 3). Skewness statistics were positive: 0.50–11.41. All variables appeared normally distributed in this study sample except for the amount of money they had spent on gambling, which was below the threshold of 3. The distribution of “the amount of money” variable was highly skewed: 11.41. We also conducted correlation analyses among gambling-related variables (Table 4). Correlation analyses demonstrated that all three variables of gambling behaviours and all five domains of gambling cognitive distortions were significantly and mildly–to–moderately correlated with gambling symptoms (gambling behaviours: *r* = .33–.58; gambling cognitive distortions: *r* = .41 –. 53). Moreover, all gambling behaviours were significantly and mildly–to–moderately correlated with gambling cognitive distortions (*r* = .20–.42).

**Table 4:** Correlation coefficients for measurements

	Behavior 1	Behavior 2	Behavior 3	Factor 1	Factor 2	Factor 3	Factor 4	Factor 5
SOGS	.58^*^	.33^*^	.36^*^	.41^*^	.45^*^	.46^*^	.53^*^	.48^*^
Gambling behavior 1	-	.52^*^	.42^*^	.31^*^	.32^*^	.39^*^	.41^*^	.42^*^
Gambling behavior 2		-	.20^*^	.21^*^	.20^*^	.20^*^	.21^*^	.25^*^
Gambling behavior 3			-	.21^*^	.27^*^	.22^*^	.34^*^	.22^*^
GRCSfactor 1				-	.74^*^	.68^*^	.67^*^	.56^*^
GRCSfactor 2					-	.84^*^	.72^*^	.59^*^
GRCSfactor 3						-	.74^*^	.68^*^
GRCSfactor 4							-	.65^*^
GRCSfactor 5								-

*SOGS* South Oaks Gambling Screen, *GRCS* Gambling Related Cognitions Scale, *p<.05, gambling behavior 1 number of days, gambling behavior 2 amount money, gambling behavior 3 gambling history, GRCSfactor 1 illusion of control, GRCSfactor 2 predictive control, GRCSfactor 3 interpretative bias, GRCSfactor 4 gambling expectancy, GRCSfactor 5 perceived inability to stop gambling

### Hierarchical multivariate regression analysis

We conducted a hierarchical multivariate regression analysis to assess the effects of gambling cognitive distortions on gambling disorder symptoms, adjusting students’ sex, age, income, and gambling behaviours (Table 5). For 342 gamblers, about 37% of the variance in gambling symptoms was explained by the measure entered at Step 1. In particular, sex (*β* = −.10, *p* < .05) and gambling behaviours (number of days *β* = .48, *p* < .05; gambling history *β* = .14, *p* < .05) were significant predictive factors of gambling symptoms. When the five domains of gambling cognitive distortions were entered in Step 2, gambling expectancy was found to be associated significantly with gambling symptoms (*β* = .19, *p* < .05). The final model explained 47% of the variance in gambling symptoms. In this regression model, the variance inflation factors were below the standard of 10.0, which indicated that multicollinearity did not present a biasing problem in the data. Moreover, we conducted a similar analysis using the data of all participants (*N* = 1471). Similar to the results for gamblers, in the final model gambling expectancy was found to be associated significantly with gambling symptoms (*β* = .19, *p* < .05). Moreover, other cognitive distortion (perceived inability to stop gambling) also was associated with gambling symptoms (*β* = .13, *p* < .05). This model explained 50% of the variance in gambling symptoms.

**Table 5:** Results of hierarchical multivariates regression analysis

	Total (*N* = 1,471)						Gambler (*N* = 342)					
	Step 1 (ΔR^2^ = 43*)			Step 2 (ΔR^2^ = 07*)			Step 1 (ΔR^2^ = 37*)			Step 2 (ΔR^2^ = 10*)		
	b	SE	β	b	SE	β	b	SE	β	b	SE	β
Sex	-.37	.07	-.11*	-.28	.07	-.08	-.51	.24	-.10*	-.38	.23	-.07
Age	.01	.03	.06	.01	.03	.01	-.04	.08	-.03	-.02	.07	-.01
Income	.00	.00	.03	.00	.00	.02	.00	.00	.01	.00	.00	.01
Gambling behavior (number of days)	.20	.01	.46*	.19	.01	.38*	.20	.02	.48*	.15	.02	.37*
Gambling behavior (amount of money)	.00	.00	.06	.00	.00	.06	.00	.00	.04	.00	.00	.03
Gambling behavior (gambling history)	.02	.00	.20*	.02	.00	.15*	.01	.00	.14*	.01	.00	.07
GRCSfactor 1 (illusion of control)				-.02	.01	-.06				.01	.03	.02
GRCSfactor 2 (predictive control)				.02	.01	.08				.04	.03	.01
GRCSfactor 3 (interpretative bias)				-.01	.01	-.04				-.02	.03	-.04
GRCSfactor 4 (gambling expectancy)				.07	.01	.19*				.08	.03	.19
GRCSfactor 5 (perceived inability to stop gambling)				.04	.02	.13*				.04	.02	.12

*SOGS* South Oaks Gambling Screen, *GRCS* Gambling Related Cognitions Scale

^*^p <.05

## Discussion

The study aimed to describe the symptoms, behaviours, and cognitive distortions of gambling in a university student sample in Japan, and to ascertain what factors of gambling cognitive distortions have predictive effects on gambling disorder symptoms.

First, for the gambling symptoms, types, and cognitive distortions in Japanese university students, 5.1% (*n* = 75) of this study’s participants were classified as probable disordered gamblers. This rate is similar to the estimated rate of Nowak’s meta-analysis (estimated rate = 6.13%; 95% CI = 5.19–7.07), which studied disordered gambler data obtained mainly from Western and English-speaking university students [20].

Furthermore, for the types of gambling that respondents had participated in during the prior year, the most common response was pachislot: 32.7%. These results indicate that the bias of the gambling type to pachislot and pachinko was a characteristic unique to gamblers in Japan that is unlike that of Western gamblers. That is because pachislot and pachinko’s stores and gambling games are the predominant type in Japan, so people have more access to and familiarity with these gambling. Pachislot is similar to slot machines games in Western casinos while pachinko resembles a vertical pinball machine.

Based on their GRCS scores, the participants in this study presented with gambling cognitive distortions similar to those of adult gamblers (sample overall score = 54.44; sample factor scores = 8.23–15.08 [18]) and treatment-seeking gamblers [5]. The results of correlation analyses and a hierarchical multivariate regression analysis revealed that increased gambling-related cognitions are related to a higher level of gambling symptoms (*r* = .41–.53). Moreover, the hierarchical regression analysis revealed that the gambling expectancy is significantly associated with gambling symptoms. These results are consistent with earlier research showing that probable disordered gamblers were more likely to make irrational predictions of gambling outcomes and had more positive gambling expectancies [7, 29]. Because disordered gamblers are likely to possess cognitive distortions [8], the inclusion of gambling cognitive distortion is crucial in the treatment of gambling disorders [6]. Earlier studies have identified risk factors of gambling disorder among university students, such as, male gender, habits of drinking and skipping breakfast, mental health difficulties, deficient social support, sociability, and neurotic personality characteristics (e.g., [28, 30]). Future studies must assess the effects of interactions between these risk factors and cognitive distortions, especially gambling expectancy and perceived inability to stop gambling, for gambling symptoms. Moreover, an earlier study [12] found that problem gamblers had more gambling cognitive distortions than non-problem gamblers. In the future, we would need to compare gambling cognitive distortions between disordered gamblers and non-problem/at-risk gamblers in Japan.

A potential limitation of this study was our sampling. We were only using 342 data from participants who scored one or more on the SOGS, which was a small percent of our all participants. In the future, replication of the results using another sample, such as internet sample and/or clinical sample is necessary. Moreover, the results of this study were based on the cross-sectional design and were found from the correlational and regression analyses. And in the regression analysis, gambling distortions explained only 10% of the variance in the final model. Therefore, we cannot infer causal relationships between gambling symptom and cognitive distortions from these data. We need to conduct the longitudinal study and the research designed based on using mediation analysis for clarifying these causal relationships in the future.

A limitation of the current study can provide useful direction for future studies. This study is the first attempt to assess gambling symptoms, types, and cognitive distortions for university students in Japan. We believe that replication of the results of this study in another university student sample would be beneficial for establishing the generalizability of our results. Moreover, a second measure now exists for assessing gambling symptoms (e.g., Gambling Symptom Assessing Scale [13]). Consequently, replication of the results using another scale measuring gambling symptoms is necessary.

## Conclusions

Despite these limitations, this study achieved an important goal contributing data to the nascent research on university student gambling in Japan. Specifically, our results indicated a relation between gambling cognitive distortions and gambling disorder symptoms, which can inform the development of preventive education and treatment for university students with gambling disorder. For example, we thought it is useful for university students gamblers in Japan to develop the preventive education and treatment focused on cognitive reconstructing for gambling expectancy.

## Data Availability

The datasets during and/or analysed during the current study available from the corresponding author on reasonable request.

## Abbreviations

GPA: Grade Point Average
GRCS: Gambling Related Cognitions Scale
SOGS: South Oaks Gambling Screen
